# Nomogram for perinatal prediction of intrapartum fever: a retrospective case–control study

**DOI:** 10.1186/s12884-021-03891-6

**Published:** 2021-06-25

**Authors:** Zhenfei Jiang, Xiaoyi Hu, Huabei Zeng, Xinghe Wang, Cheng Tan, Chunyan Ni, Lingyun Dai, Su Liu

**Affiliations:** 1 Jiangsu Province Key Laboratory of Anesthesiology, Xuzhou Medical University, Xuzhou, Jiangsu China; 2 Department of Anesthesiology, Affiliated Hospital of Xuzhou Medical University, Xuzhou, 221000 Jiangsu China; 3 Department of Anesthesiology, Obstetrics and Gynecology Hospital, Suqian, Jiangsu, China; 4 Department of Anesthesiology, Suqian First People’s Hospital, Jiangsu, China

**Keywords:** Intrapartum fever, Risk factors, Predictive model, Nomogram

## Abstract

**Objective:**

To explore the risk factors for intrapartum fever and to develop a nomogram to predict the incidence of intrapartum fever.

**Methods:**

The general demographic characteristics and perinatal factors of 696 parturients who underwent vaginal birth at the Affiliated Hospital of Xuzhou Medical University from May 2019 to April 2020 were retrospectively analysed. Data was collected from May 2019 to October 2019 on 487 pregnant women who formed a training cohort. A multivariate logistic regression model was used to identify the independent risk factors associated with intrapartum fever during vaginal birth, and a nomogram was developed to predict the occurrence. To verify the nomogram, data was collected from January 2020 to April in 2020 from 209 pregnant women who formed a validation cohort.

**Results:**

The incidence of intrapartum fever in the training cohort was found in 72 of the 487 parturients (14.8%), and the incidence of intrapartum fever in the validation cohort was 31 of the 209 parturients (14.8%). Multivariate logistic regression analysis showed that the following factors were significantly related to intrapartum fever: primiparas (odds ratio [OR] 2.43; 95% confidence interval [CI] 1.15–5.15), epidural labour analgesia (OR 2.89; 95% CI 1.23–6.82), premature rupture of membranes (OR 2.37; 95% CI 1.13–4.95), second stage of labour ≥ 120 min (OR 4.36; 95% CI 1.42–13.41), amniotic fluid pollution degree III (OR 10.39; 95% CI 3.30–32.73), and foetal weight ≥ 4000 g (OR 7.49; 95% CI 2.12–26.54). Based on clinical experience and previous studies, the duration of epidural labour analgesia also appeared to be a meaningful factor for intrapartum fever; therefore, these seven variables were used to develop a nomogram to predict intrapartum fever in parturients. The nomogram achieved a good area under the ROC curve of 0.86 and 0.81 in the training and in the validation cohorts, respectively. Additionally, the nomogram had a well-fitted calibration curve, which also showed excellent diagnostic performance.

**Conclusion:**

We constructed a model to predict the occurrence of fever during childbirth and developed an accessible nomogram to help doctors assess the risk of fever during childbirth. Such assessment may be helpful in implementing reasonable treatment measures.

**Trial registration:**

Clinical Trial Registration: (www.chictr.org.cnChiCTR2000035593)

## Introduction

Intrapartum fever, which is defined as a maternal body temperature of greater than or equal to 38℃ during labour and childbirth. The prevalence of intrapartum fever ranges from 1.6% to 14.6% [1]. Previously, intrapartum fever was believed to be the result of an infectious inflammation in the parturient, but administration of the antibiotic cefonicid did not reduce the occurrence of intrapartum fever [2]. This finding suggests that intrapartum fever cannot be completely attributed to maternal infection. Current research suggests that most intrapartum fever is secondary to noninflammatory infection [3, 4].

A few studies have shown that intrapartum fever has serious adverse consequences on maternal safety and on newborn growth and development. Women with maternal fever are more likely to receive antibiotics and undergo caesarean Sect [5]. Additionally, maternal fever may be associated with low Apgar scores, respiratory distress, hypotonia, and neonatal seizures [6–8]. Of serious consequence, intrapartum fever has been found to be related to neonatal encephalopathy [9]. The long-term prognosis of children with neonatal encephalopathy depends on its severity, and may cause cerebral palsy and mental retardation [10]. The current research on the risk factors for intrapartum fever has been studied, but the studies have been limited by small sample sizes or missing data [11, 12]. In addition, the previous research does not distinguish between low-risk and high-risk parturients. If the risk and incidence of fever during delivery can be predicted early and accurately, it may help obstetricians provide intervention measures. Such measures might include increasing the monitoring of body temperature during childbirth, minimizing labour time, reducing the number of vaginal examinations, weighing the advantages and disadvantages of artificial membrane rupture, using oxytocin and antibiotics in advance, so as to better managing the stage of labour.

Owing to the lack of a specific and practical predictive method, the development of a prediction model that incorporates factors associated with intrapartum fever based on perinatal clinical data is desirable. Of all available models, nomograms can provide a personalized, evidence-based, and highly accurate risk estimation [13]. The nomogram is easy to use and can guide relevant clinical management. To the best of our knowledge, no such model has been established to help identify women at high risk of intrapartum fever.

Therefore, the objective of this study was to conduct a comprehensive and systematic review of antenatal and perinatal factors related to intrapartum fever in order to distinguish independent risk factors for perinatal fever. A risk prediction model was established, and a nomogram was developed to assist obstetricians in identifying clinically high-risk women and in optimizing management in the early stages of labour.

## Methods

This retrospective case–control study was registered in the Chinese Clinical Trial Center (ChiCTR2000035593) and approved by the Ethics Committee of the Affiliated Hospital of Xuzhou Medical University (XYFY2020-KL135-01). Informed consent was obtained over the phone and verbal consent from all mothers for their data to be used for research. Data were abstracted on women who underwent a vaginal birth at the Affiliated Hospital of Xuzhou Medical University from May 2019 to April 2020. The inclusion criteria included a singleton pregnancy, cephalic presentation, term birth (37–41 weeks of gestation), and vaginal birth. The exclusion criteria included women likely to be in a hypermetabolic state with basal body temperatures ≥ 37.5℃, women who had other known infections, and parturients who were underwent a caesarean section for non-febrile reasons (these women were mostly multiparous who had a caesarean section (CS) before or had complications such as placenta previa and early placement of the placenta and some women requested caesarean section by themselves, information of labor time, time of membrane rupture, number of vaginal examinations and so on of these people were not recorded). Women who experienced a premature birth or whose electronic medical records were missing were also excluded.

All mothers and infants received standard obstetric and neonatal care. If the woman chose to use epidural anaesthesia, the epidural analgesia pump regimen remained unchanged (PCEA: loading dose: 10 ml 0.1% ropivacaine + 0.5 µg/ml sufentanil; maintenance: 0.1% ropivacaine + 0.5 µg/ml sufentanil at 8 ml/h; bolus 8 ml; lock-out 30 min). It remained up to the obstetricians whether to use antibiotics or antipyretic therapy during labour.

Based on relevant literature and expert opinions, [11, 12] the following factors were collected from the electronic medical record system as observation indicators: general demographic characteristics including maternal age, BMI, gestational age, parity, and foetal weight; perinatal factors including body temperature on admission, white blood cell count on admission, haemoglobin on admission, pregnancy complications (gestational diabetes, hypertension during pregnancy, abnormal thyroid function), pre-labour rupture of membrane (pre-labour ROM), duration from rupture of membranes to childbirth, duration of the first and second stages of labour, epidural labour analgesia, duration of analgesia, amniotic fluid pollution degree III (defined as yellowish-brown, viscous amniotic fluid combined with yellowish foetal membranes), oxytocin usage, and the number of vaginal examinations.

### Statistical analysis

Using IBM SPSS 23.0 software for statistical analysis, numeric variables were analysed for a normal distribution by the Shapiro–Wilk test. Continuous variables with a normal distribution were expressed as the mean ± standard deviation (SD) and were compared using the independent-sample *t*-test. Continuous variables with a nonnormal distribution were expressed as the median (interquartile range), and comparisons between groups were presented by the Mann–Whitney *U-*test. Categorical data were presented as a number (%) and were analysed using the χ^2^ test or Fisher's exact probability test. Missing data were analysed using the mean interpolation method. The significance of each variable in the training cohort was assessed by univariate analysis to investigate the independent factors of intrapartum fever. All variables associated with intrapartum fever at a significant level were candidates for stepwise multivariate logistic regression analysis using the stepwise variable selection method. All potential predictors were included. The standard for entering multivariate analysis was *P* < 0.2, and retention in the logistic regression model required *P* < 0.05. The results were expressed as odds ratio (OR) and 95% CIs.

R4.0.3 software was used to develop a predictive model and draw a nomogram to predict the occurrence of intrapartum fever [14]. Discrimination and calibration were used to verify the predictive ability of the model. Discrimination was expressed by the area under the receiver operating characteristic curve, and the Youden index (sensitivity + specificity -1) was used to find the best critical value (Cut-off value). The accuracy of the best Cut-off value was evaluated by sensitivity, specificity, predictive value, and likelihood ratios. Calibration was demonstrated by the Hosmer–Lemeshow goodness-of-fit test, which compares the difference between the predicted probability and the actual probability. A calibration plot was drawn from these data. *P* > 0.05 indicates that the difference between the predicted value and the actual probability of the outcome was not statistically significant, which represents goodness of fit.

## Results

**Fig. 1:**
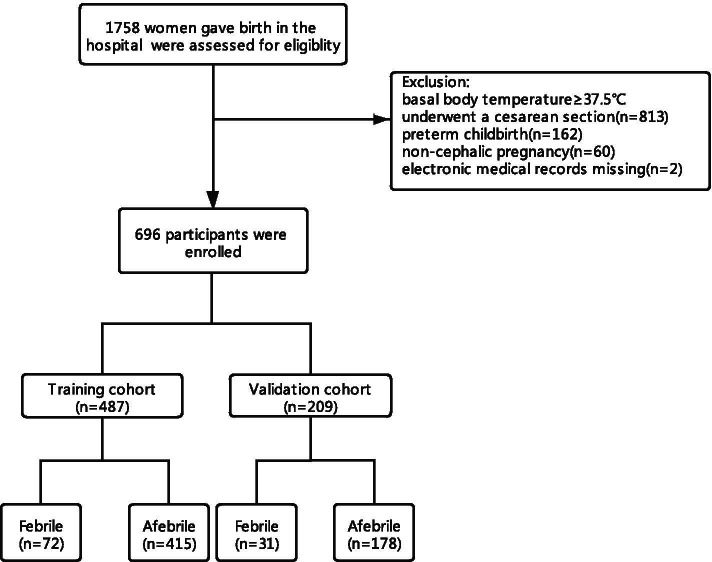
The Participants recruitment flowchart

From May 2019 to April 2020, 1,762 pregnant women planned a vaginal birth at the Affiliated Hospital of Xuzhou Medical University. These parturients were potential participants in a study conducted for the purpose of developing a nomogram to identify parturients at risk of developing intrapartum fever. After exclusions and eliminations, 696 parturients were included in the study. Four hundred eighty-seven participants formed a training cohort. The data collected from these participants from May 2019 to December 2019 were used to assess the risk factors for intrapartum fever and to develop the nomogram. The women were divided into an afebrile group (*n* = 415) and a febrile group (*n* = 72) according to whether or not their intrapartum body temperature exceeded 38 °C. The remaining 209 women formed a validation cohort. The data collected from these participants were abstracted from January 2020 to April 2020 for the purpose of testing the newly developed nomogram. The Participants recruitment flowchart is shown in Fig. 1.

Table 1 presents the general demographic characteristics and perinatal factors of the training and the validation cohorts. No statistically significant difference was found between the two groups.

**Table 1:** Comparison of demographic characteristics and perinatal factors between the training and the validation cohorts

Variable	Training cohort (*n* = 487)	Validation cohort (*n* = 209)	*P*
Age [M (IQR)]	28 (4)	29 (5)	0.081
BMI, kg/m^2^ [M (IQR)]	26.7 (3.9)	26.4 (4.7)	0.565
Gestational age, d [M (IQR)]	275 (11)	274 (10)	0.402
Accompany disease, n (%)			
GDM	62 (12.7%)	2 4(11.5%)	0.647
Abnormal thyroid function	8 (1.6%)	3 (1.4%)	0.841
Hypertension during pregnancy	20 (4.2%)	10 (4.8%)	0.729
Parity, n (%)			0.708
Nulliparity	294 (60.4%)	123 (58.9%)	
Multiparous	193 (39.6%)	86 (41.1%)	
Body temperature on admission, ℃ [M (IQR)]	36.5 (0.2)	36.5 (0.2)	0.232
WBC counts on admission, ×10^9^ [M (IQR)]	8.3 (2.7)	8.7 (3.0)	0.168
HB on admission, g/L [M (IQR)]	12.1 (2)	12 (2)	0.508
Pre-labour ROM, n (%)			0.324
Yes	72 (14.8%)	25 (12%)	
No	415 (85.2%)	184 (88%)	
Duration from rupture of membranes to delivery, min [M (IQR)]	188 (434)	234 (480)	0.836
Method of membrane rupture, n (%)			0.407
Spontaneous	224 (46.0%)	89 (42.6%)	
Surgical	263 (54%)	120 (57.4%)	
Oxytocin usage, n (%)			0.325
Yes	276 (56.7%)	110 (52.6%)	
No	211 (43.3%)	99 (47.4%)	
Foetal weight, g [M (IQR)]	3340 (490)	3350 (580)	0.749
Amniotic fluid pollution III, n (%)			0.686
Yes	20 (4.1%)	11 (5.3%)	
No	467 (95.9%)	198 (94.7%)	
Duration of the first stage of labour, min [M (IQR)]	470 (450)	420 (438)	0.203
Duration of the second stage of labour, min [M (IQR)]	31 (46)	28 (48)	0.120
Number of vaginal examinations [M (IQR)]	2 (1)	2 (2)	0.803
Epidural labour analgesia, n (%)			0.933
Yes	139 (28.5%)	59 (28.2%)	
No	348(71.5%)	150 (71.8%)	
Analgesia time, min [M (IQR)]	0(160)	0 (133)	0.834
Apgar score at 5 minutes [M (IQR)]	9(10)	9 (10)	0.956

*Abbreviations*: *BMI* body mass index, *GDM* gestational diabetes, *HB* haemoglobin, *IQR* interquartile range, *M* median, *ROM* rupture of membrane, *WBC* white blood cell

The data on 487 parturients in the training cohort were analysed to examine the influencing factors of intrapartum fever. The univariate analysis results of the influencing factors related to intrapartum fever are shown in Table 2. The results showed that nulliparity, pre-labour rupture of membranes, duration from rupture of membranes to childbirth, foetal weight, method of membrane rupture, oxytocin usage, amniotic fluid pollution III, duration of the first stage of labour, duration of the second stage of labour, number of vaginal examinations, epidural labour analgesia, and analgesia time were related to intrapartum fever (*P* < 0.2).

**Table 2:** Univariate analysis of factors related to intrapartum fever (training cohort)

	Febrile (*n* = 72)	Afebrile (*n* = 415)	*P*
Age [M (IQR)]	28 (4)	28 (4)	0.760
BMI, kg/m^2^ [M (IQR)]	26.3 (3.8)	26.8 (3.9)	0.756
Gestational age, d [M (IQR)]	275.5 (11)	275 (11)	0.466
Accompany disease, n (%)			
GDM	10 (13.9%)	52 (12.5%)	0.749
Abnormal thyroid function	3 (4.2%)	14 (3.5%)	0.667
Hypertension during pregnancy	1 (1.4%)	7 (1.7%)	0.854
Parity, n (%)			< 0.001
Nulliparity	59 (81.9%)	235 (56.6%)	
Multiparous	13 (18.1%)	180 (43.4%)	
Pre-labour ROM, n (%)			0.001
Yes	20 (27.8%)	52 (12.5%)	
No	52 (72.2%)	363 (87.5%)	
Body temperature on admission,℃ [M (IQR)]	36.5 (0.2)	36.5 (0.2)	0.414
WBC counts on admission, × 10^9^ [M (IQR)]	7.95 (3.2)	8.4 (2.5)	0.360
HB on admission, g/L [M (IQR)]	12.05 ± 1.24	12.07 ± 1.1	0.995
Time from rupture of membranes to delivery, min [M (IQR)]	318.5 (671)	168 (406)	0.009
Method of membrane rupture, n (%)			0.078
Spontaneous	40 (55.6%)	184 (44.3%)	
Surgical	32 (44.4%)	231 (55.7%)	
Oxytocin usage, n (%)			0.004
Yes	52 (72.2%)	224 (54%)	
No	20 (27.8%)	191 (46%)	
Foetal weight, g [M (IQR)]	3410 (508)	3320 (480)	0.175
Amniotic fluid pollution degree III, n (%)			< 0.001
Yes	12 (16.6%)	8 (1.9%)	
No	60 (83.4%)	407 (98.1%)	
Duration of the first stage, min [M (IQR)]	695.9 (192.3)	417.5 (443.1)	< 0.001
Duration of the second stage, min [M (IQR)]	83.8 (48.5)	28 (38)	< 0.001
Number of vaginal examinations [M (IQR)]	3 (2)	2 (2)	< 0.001
Epidural labour analgesia, n (%)			< 0.001
Yes	50 (69.4%)	89 (21.4%)	
No	22 (30.6%)	326 (78.6%)	
Analgesia time, min [M (IQR)]	330 (433)	0 (100)	< 0.001

*Abbreviations*: *BMI* Body mass index, *GDM* Gestational diabetes, *HB* Haemoglobin, *IQR* Interquartile range, *M* Median, *ROM* Rupture of membrane, *WBC* White blood cell

The 12 factors with significant univariate analysis results were assigned and then included in the multivariate logistic regression analysis using forward stepwise regression. The results are shown in Table 3.

**Table 3:** Multivariate analysis of factors related to intrapartum fever

Risk factor	B	SE	Wald	*P*	OR	95% CI
Nulliparity	0.89	0.38	5.40	0.02	2.43	1.15–5.15
Pre-labour ROM	0.86	0.38	5.21	0.02	2.37	1.13–4.95
Epidural labour analgesia	1.06	0.44	5.87	0.02	2.89	1.23–6.82
Amniotic fluid pollution degree III	2.34	0.59	15.99	0.00	10.39	3.30–32.73
The second stage of the labour ≥ 120 min	1.47	0.57	6.61	0.01	4.36	1.42–13.41
Foetal weight	2.01	0.65	9.74	0.00	7.49	2.12–26.54
Analgesia time < 4 h			11.82	0.00		
Analgesia time (4–6 h)	-0.82	0.56	2.51	0.14	0.44	0.15–1.32
Analgesia time > 6 h	0.80	0.52	2.36	0.12	2.22	0.80–6.14

*Abbreviation*s: *B* Beta, *CI* Confidence interval, *OR* Odds ratio, *ROM* Rupture of the membrane, *SE* Standard error

Multivariate logistic regression analysis with results reported as OR (95% CI), nulliparity [2.43(1.15–5.15)], epidural labour analgesia [2.89(1.23–6.82)], pre-labour ROM [2.37(1.13–4.95)], second stage of labour ≥ 120 min [4.36(1.42–13.41)], amniotic fluid pollution degree III [10.39(3.30–32.73)], and foetal weight ≥ 4000 g [7.49(2.12–26.54)] were significantly related to intrapartum fever. Based on clinical experience and previous studies, [12, 13] the duration of epidural analgesia also played a significant role in intrapartum fever. In our study, this variable did not show any significance, but we still incorporated it into the development of the nomogram.

**Fig. 2:**
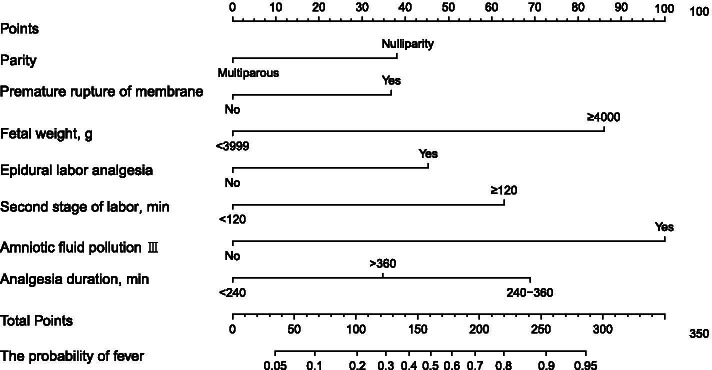
Nomogram for estimation of intrapartum fever

Figure 2 shows the nomogram formed to predict the risk of intrapartum fever based on these selected parameters.

To use the nomogram, find the position of each variable on the corresponding axis, draw a line to the points axis for the number of points, add the points from all of the variables, and draw a line from the total points axis to determine the intrapartum fever probabilities at the lower line of the nomogram.

The nomogram demonstrated good accuracy in estimating the risk of intrapartum fever, with an AUC of 0.86 (95% CI 0.81–0.90). In addition, the Hosmer–Lemeshow goodness-of-fit test (*X*^2^ = 4.585, *P* = 0.80) and calibration plots graphically indicated a good level of agreement between the predicted value of the model and the actual observed value.

**Fig. 3:**
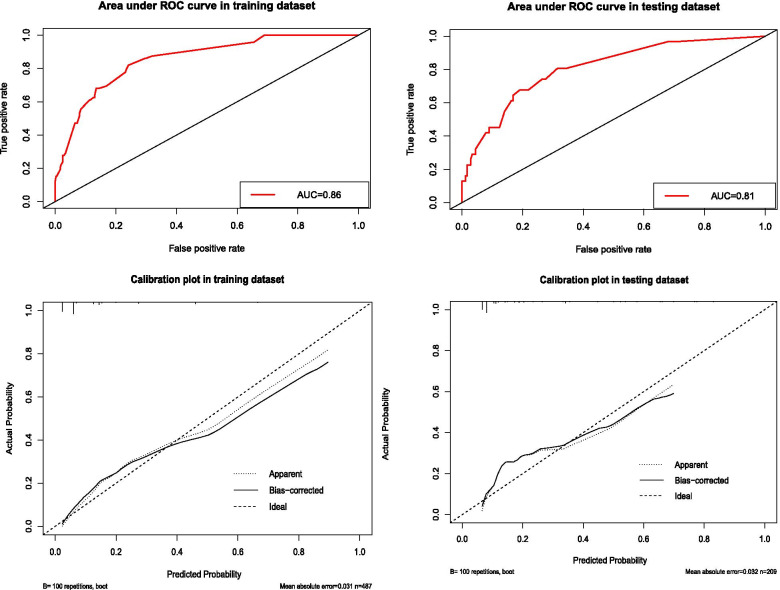
ROC curves and calibration plots for the training cohort and validation cohort. Abbreviations: ROC, receiver operating characteristic; AUC, area under the ROC curve

In the validation cohort, the nomogram displayed an AUC of 0.81 (95% CI 0.73–0.90), and the risk estimate also had a good calibration curve. The ROC curve and calibration diagram of the training cohort and the verification cohort are shown in Fig. 3.

The best cut-off value for the total score of the nomogram was determined to be 167. The sensitivity, specificity, positive predictive value, and negative predictive value used to distinguish the occurrence of intrapartum fever were 88.6%, 66.7%, 97.6%, and 27.8% in the training cohort and 88.5%, 52.9%, 95.5% and 29.0% in the validation cohort, respectively (Table 4).

**Table 4:** Accuracy of the prediction scores of the nomogram for estimating the risk of intrapartum fever

Variable	Value (95% CI)	
	Training cohort	Validation cohort
Area under ROC curve (CI)	0.86 (0.81–0.90)	0.81 (0.73–0.90)
Cut-off score	167	167
Sensitivity, %	88.6 (85.3–91.3)	88.5 (83.0–92.5)
Specificity, %	66.7 (47.1–82.1)	52.9 (28.5–76.1)
Positive predictive value, %	97.6 (95.5–98.8)	95.5 (91.0–97.9)
Negative predictive value, %	27.8 (18.2–39.8)	29.0 (14.9–48.2)
Positive likelihood ratio	2.66 (1.60–4.42)	1.88 (1.13–3.12)
Negative likelihood ratio	0.17 (0.13–0.23)	0.22 (0.13–0.36)

*Abbreviations*: *CI* Confidence interval, *ROC* Receiver operating characteristic

Perinatal outcomes of mothers and infants in the febrile and the afebrile groups were also investigated. The results confirmed that maternal fever during delivery increased the rate of caesarean delivery, the amount of bleeding during labour, and increased the chance of antibiotic use. Because only Apgar scores were recorded in the obstetrical records, we only analysed Apgar scores < 7 at 5 min, and we found that the febrile group had lower Apgar scores for newborns (*P* < 0.05) (Table 5).

**Table 5:** Comparison of maternal and infant perinatal outcomes between the febrile and the afebrile groups

	Febrile (*n* = 104)	Afebrile (*n* = 593)	*P*
Transferred to caesarean delivery	27 (26%)	0 (0%)	< 0.001
Bleeding during delivery	250 (100)	300 (200)	< 0.001
Antibiotic			< 0.001
Yes	65 (90.3%)	106 (25.5%)	
No	7 (9.7%)	309 (74.5%)	
Indometacin	18(17.3%)	0(0%)	< 0.001
Paracetamol	6(5.8%)	0(0%)	< 0.001
Apgar score < 7 at 5 min	6 (5.8%)	4 (0.7%)	0.001

## Discussion

In this study, we systematically analysed the risk factors for intrapartum fever, and we established a predictive model that incorporated the following seven factors into its construction: nulliparity, prelabour rupture of membranes, foetal weight, epidural analgesia during labour, duration of the second stage of labour ≥ 120 min, amniotic fluid pollution degree III, and duration of epidural analgesia. The predictive model was represented by a nomogram. To the best of our knowledge, this is the first nomogram used to distinguish high-risk puerperae who may experience intrapartum fever.

Intrapartum fever is a common complication during labour and childbirth [15]. The incidence of intrapartum fever in our study was 14.8%, which is similar to that found in previous studies [1]. The mechanism may be endogenous heat generated by contractions of the uterus and skeletal muscle, infectious inflammation after rupture of the amniotic membrane, or epidural analgesia [16, 17]. Intrapartum fever has a close relationship with adverse outcomes of mothers and newborns. Therefore, we collected maternal-related perinatal data and confirmed the independent risk factors for intrapartum fever through multivariate logistic regression, including nulliparity, pre-labour rupture of membranes, foetal weight, epidural analgesia during labour, duration of the second stage of labour ≥ 120 min, and amniotic fluid pollution degree III. The risk factors identified above for intrapartum fever have been noted in prior studies [11, 12, 18]. Interestingly, some other factors examined like BMI, longer premature rupture of membranes, and more frequent vaginal examinations were not independent risk factors for fever during labour, and gestational age, body temperature on admission, WBC on admission, HB on admission, etc., did not show significant difference. We suppose that this may be due to the small sample size or puerperae of different regions having different physiological characteristics; for example, the timing of vaginal examinations is different among different health centres.

It is well known that pregnancy is similar to the immune response of sterile inflammation in many aspects. It has been confirmed that pregnancy is accompanied by increased inflammation. [19, 20]. Pregnancy related inflammation is also considered to likely be the pathophysiological basis of intrapartum fever. This finding is supported in Riley’s study [21]. Regardless of whether intrapartum fever occurs or not, if the parturient receives epidural analgesia during delivery, the levels of IL-6 and IL-8 are significantly higher than levels of these factors at admission, and this phenomenon is not related to infection. In addition, Riley et al. also pointed out that intrapartum fever is more likely to occur in women who have higher levels of IL-6 and IL-8 before receiving epidural analgesia. However, Arce collected blood samples at 9.7, 17.9, 26, and 35.1 weeks of pregnancy to investigate the levels of inflammatory factors during pregnancy. The results showed that the occurrence of intrapartum fever is not significantly related to the level of any inflammatory factors at any time point in pregnancy [22]. This suggests that the level of inflammation before labour was not a predictor of the occurrence of intrapartum fever, which is in agreement with our findings. It is possible that the onset of intrapartum fever is not dependent on prenatal inflammatory levels but is more likely to be triggered by events during labour that enhance maternal inflammatory responses. Our hospital did not routinely measure the level of inflammation during childbirth; thus, further studies are needed to explore the relationship between maternal fever, the level of maternal inflammation, and the origin of inflammation levels.

Epidural labour analgesia has been found to be an independent risk factor for intrapartum fever since 1994; its prevalence ranges from 1.6% to 46.3%, with an average of approximately 20% [23]. This phenomenon is known as epidural-related maternal fever (ERMF). Studies have shown that ERMF accounts for 90.4% of fever in full-term pregnancies. The rate of caesarean section and instrumental assisted birth was also significantly higher in women receiving epidural labour analgesia than that of women not receiving this treatment [24]. At present, the most concerning mechanism of ERMF is the aseptic inflammatory mechanism [25]. The current difficulties with ERMF continue to be its identification with infectious fever and the maternal and neonatal outcomes caused by epidural analgesia. It has been reported that the time of fever is mainly concentrated at 4–6 h after epidural analgesia and that most fevers recede within 24 h, [26, 27]. Although the duration of epidural analgesia did not show a significant difference in the multivariate analysis of the current study, this may be due to the small sample size, we still included it as a risk factor in the nomogram. The ROC curve and the calibration plot both showed good performance; thus, we conclude that the duration of analgesia plays an important role in intrapartum fever.

The use of nomograms in estimating the risk of intrapartum fever is a new concept [28, 29]. The nomogram we established in this study included seven variables that can be available during labour. Our nomogram performed well, with AUCs of 0.86 and 0.81 in the training and verification cohorts, respectively. The calibration plots showed good agreement between the prediction and actual observation. For the clinical application of this model, we summarized the sensitivity, specificity, negative predictive value, and positive predictive value of using 167 as the Cut-off value in estimating intrapartum fever (Table 5). Women with a score of 167 or more are considered to be at high-risk for developing intrapartum fever. Based on this preoperative prediction, the nomogram can be used as a tool in randomized clinical trials to select women for evaluation of the efficacy of diagnosis in parturients at risk for intrapartum fever.

Although some variables like the duration of analgesia time can only be collected near the end of the labour, but with the progress of labour, when women occur these risk factors successively, if the nomogram score reaches 167, the mother would have a higher risk of developing intrapartum fever, at this moment, nursing staff and obstetricians should pay more attention to the temperature monitoring of the pregnant women.

The optimal time and decision of women who are to receive antibiotic administration still remains uncertain and challenging. Intrapartum antibiotic administration can also have serious consequences for both mothers and newborns, which can increase the NICU admission, an unnecessary work-up for sepsis and increase the fertility costs [30]. But under the guidance of the nomogram, we can identify the risk indicators and deal with them symptomatically and timely so as to reduce the use of antibiotics and ensure the safety of mothers and babies.

There were several limitations in this study. This was a retrospective study involving a small number of women and there were some inherent shortcomings with this study. Doctors and nurses did not record maternal and foetal information well, which resulted in the collection of incomplete information. Additionally, this analysis was limited to the data of a single institution. It will therefore be necessary to verify the results in other research centres. Finally, although the nomogram obtained good predictive accuracy, prospective studies are needed to further confirm the reliability of the nomogram.

## Conclusion

In this retrospective study, we established a mathematical prediction model that included seven risk factors for intrapartum fever and developed a nomogram that can be used to score the risk of fever for individual women. This nomogram can help obstetricians predict and possibly prevent intrapartum fever in pregnancy women, thus improving the labour process and outcome. Although the nomogram has certain predictive value, further comprehensive analysis and dynamic monitoring are needed for maternal and neonatal safety.

## Data Availability

The datasets used and analyzed during the current study are available from the corresponding author on reasonable request.

## Abbreviations

B: Beta
BMI: Body mass index
CI: Confidence Interval
ERMF: Epidural related maternal fever
GDM: Gestational diabetes mellitus
HB: Haemoglobin
IQR: Interquartile range
M: Median
OR: Odds ratio
ROC: Receiver operating characteristic
ROM: Rupture of membrane
SE: Standard error
WBC: White blood cell
